# Supplementary material for: Discovery of Two Inhibitors of the Type IV Pilus Assembly ATPase PilB as Potential Antivirulence Compounds
Source: Microbiol Spectr. 2022 Nov 15;10(6):e03877-22. doi: 10.1128/spectrum.03877-22 (PMC9769694; doi:10.1128/spectrum.03877-22)
Supplement: Supplemental file 1 — Supplemental material. Download spectrum.03877-22-s0001.pdf, PDF file, 0.9 MB [file spectrum.03877-22-s0001.pdf]

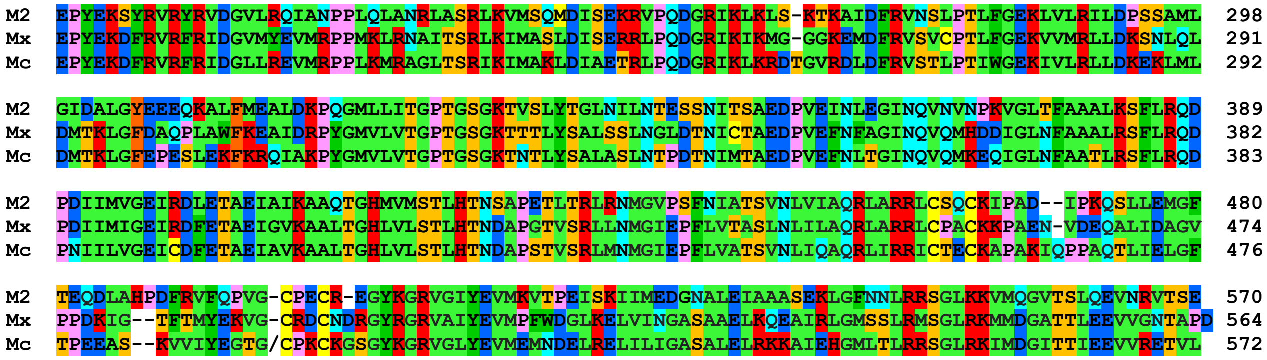

Supplemental Figure 1. Alignment of the ATPase domains of *A. nosocomialis* (M2) PilB, *M. xanthus* (Mx) PilB, and *C. thermophilum* (Mc) PilB. Residues are colored by charge, polarity, and hydrophobicity.

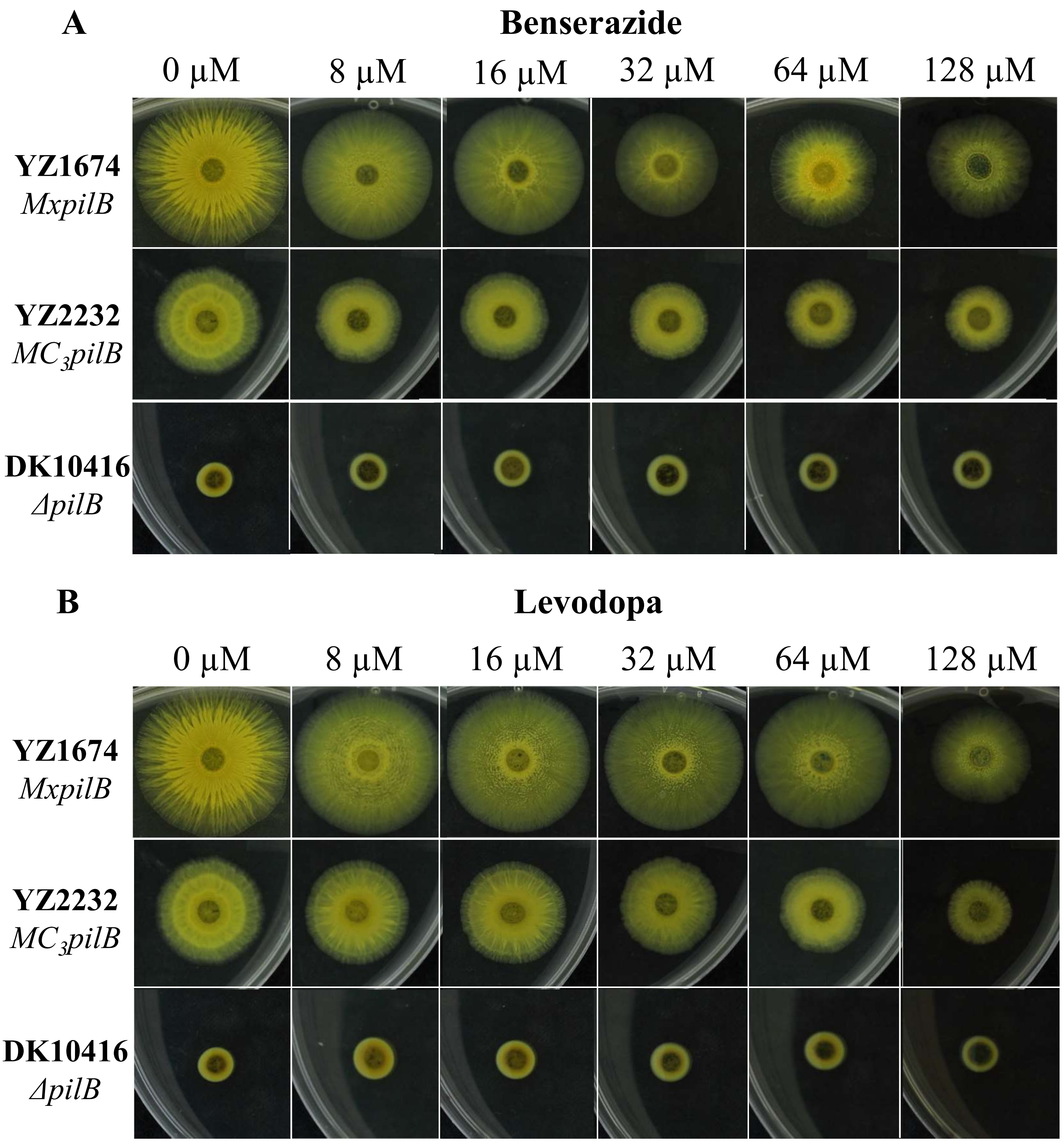

Supplemental Figure 2. Benserazide and levodopa inhibit *M. xanthus* colony expansion by T4P-dependent motility. 5 μl of cell suspension of YZ1674 (*Mx*PilB), YZ2232 (*MC*$_3$PilB), and DK10416 (Δ*pilB*) were placed on soft agar plates containing either (**A**) benserazide or (**B**) levodopa at the specified concentrations. Photographs of plates were taken after 5 days of incubation at 32°C. See text for details of the *M. xanthus* strains.

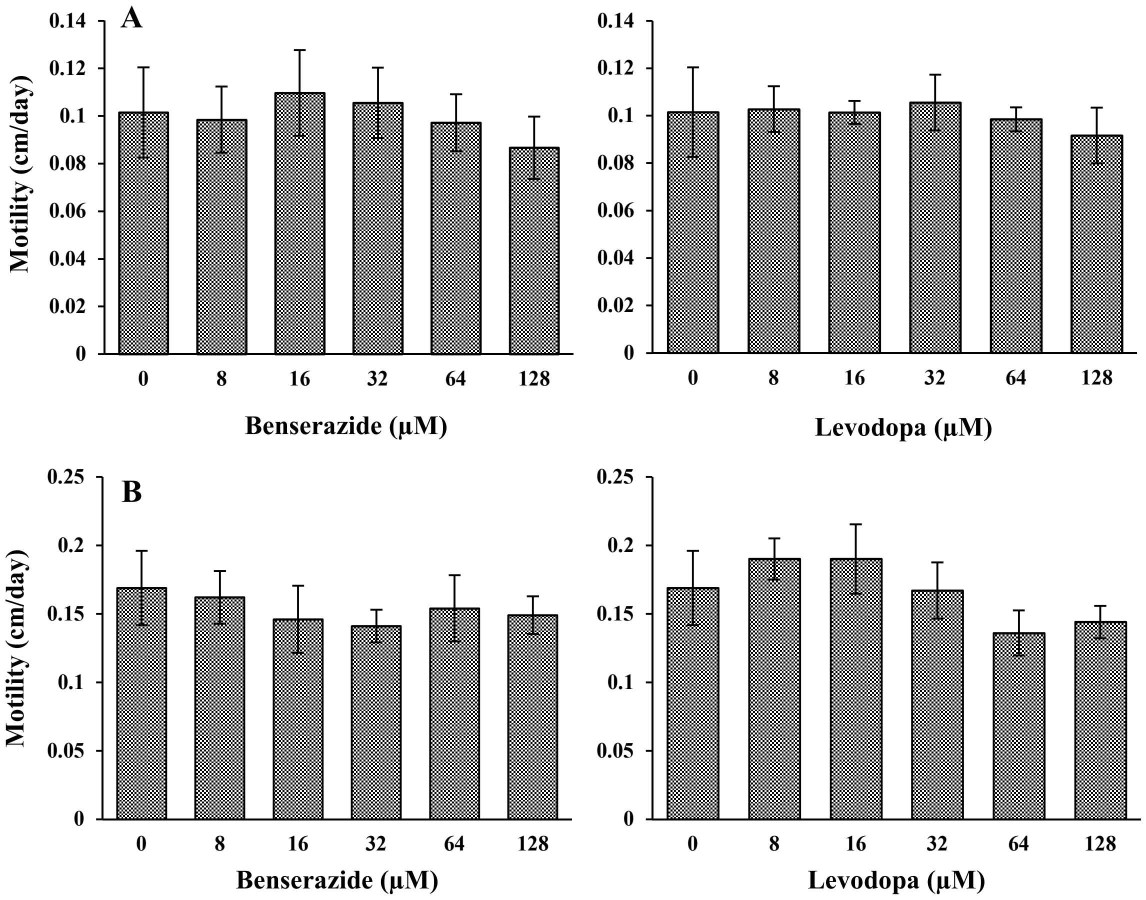

Supplemental Figure 3. Benserazide and levodopa have no effect on the rate of expansion of DK10416. (**A**) The rate of expansion of the Δ*pilB* strain (DK10416) on soft agar plates supplemented with benserazide and levodopa. (**B**) The rate of expansion of the Δ*pilB* strain on hard agar plates supplemented with benserazide and levodopa. Shown are the averages of colony expansion calculated from the measurements of at least 15 colonies at a given concentration of benserazide or levodopa. Standard deviations are represented as error bars.

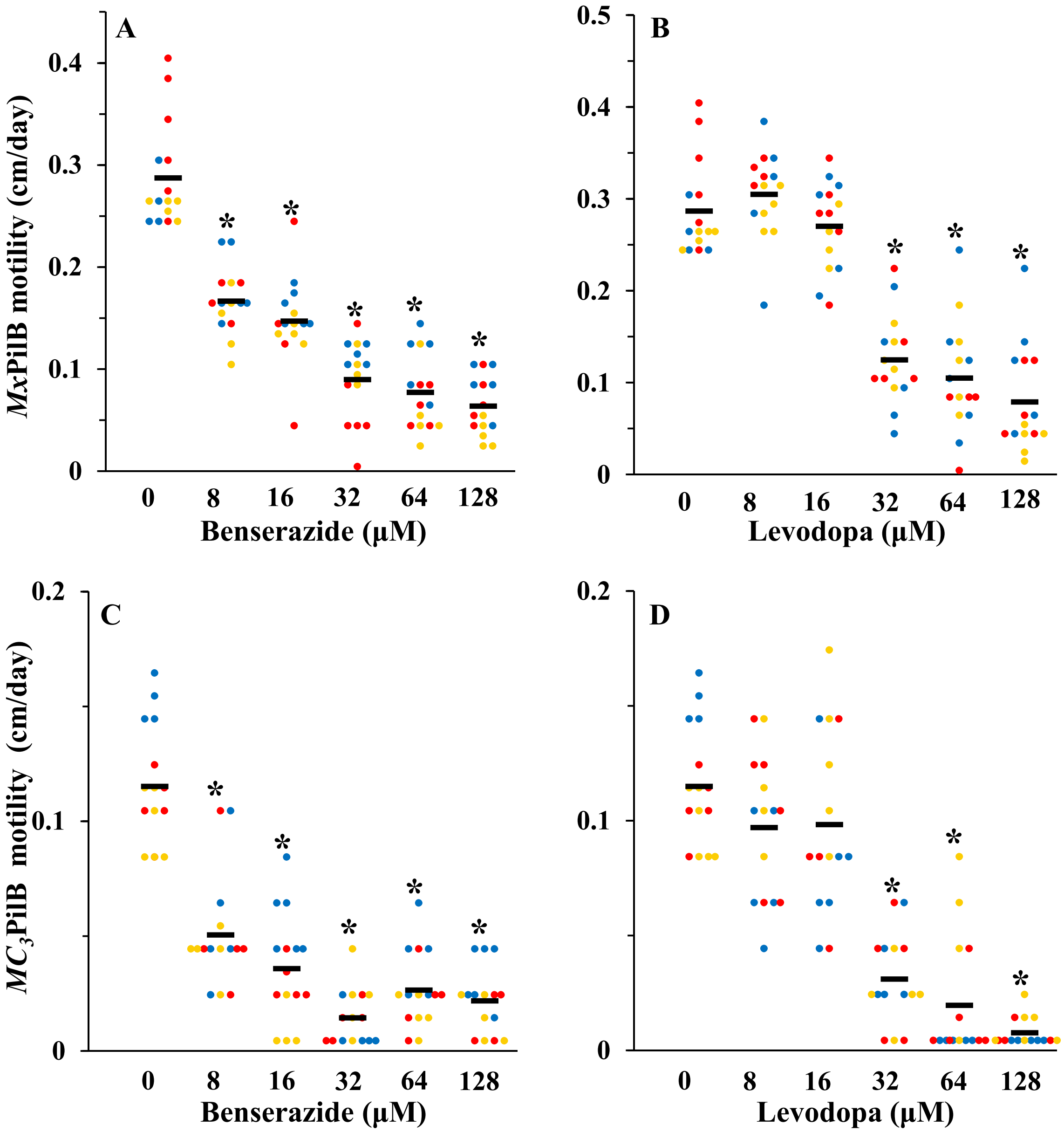

Supplemental Figure 4. Benserazide and levodopa inhibit T4P-medaited motility in *M. xanthus* on hard agar. The normalized rate expansion of the *Mx*PilB (YZ1674) (**A** & **B**) and *MC*$_3$PilB (YZ2232) (**C** & **D**) strains on hard agar plates supplemented with benserazide and levodopa. Shown are the results from three independent experiments, each represented by circles of the same color, with the black lines representing the averages. The average for a given strain was calculated from the measurements of a minimum of 15 independent colonies at a given concentration of benserazide or levodopa. The rates of expansion were normalized as described in Fig. 5. Asterisks signify that the values at the indicated concentrations are statistically different from the untreated control, with *P* values of <0.05 from Student's *t* test.

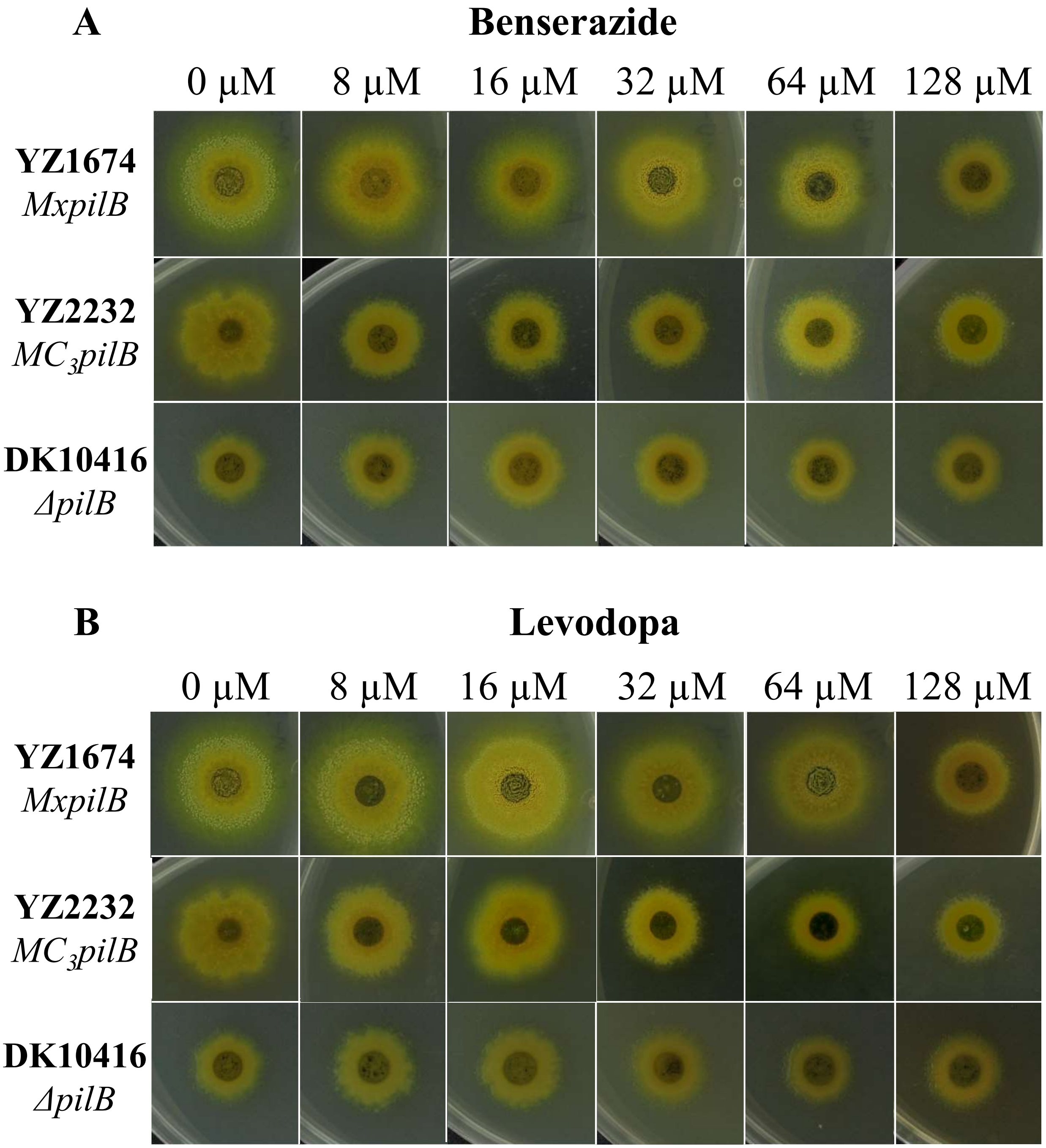

Supplemental Figure 5. Benserazide and levodopa have no effect on *M. xanthus* T4P-independent motility. 5 µl of cell suspension of YZ1674 (*Mx*PilB), YZ2232 (*MC*$_3$PilB), and DK10416 (Δ*pilB*) were placed on hard agar plates containing either (**A**) benserazide or (**B**) levodopa at the specified concentrations. Photographs of plates were taken after 5 days of incubation at 32°C.

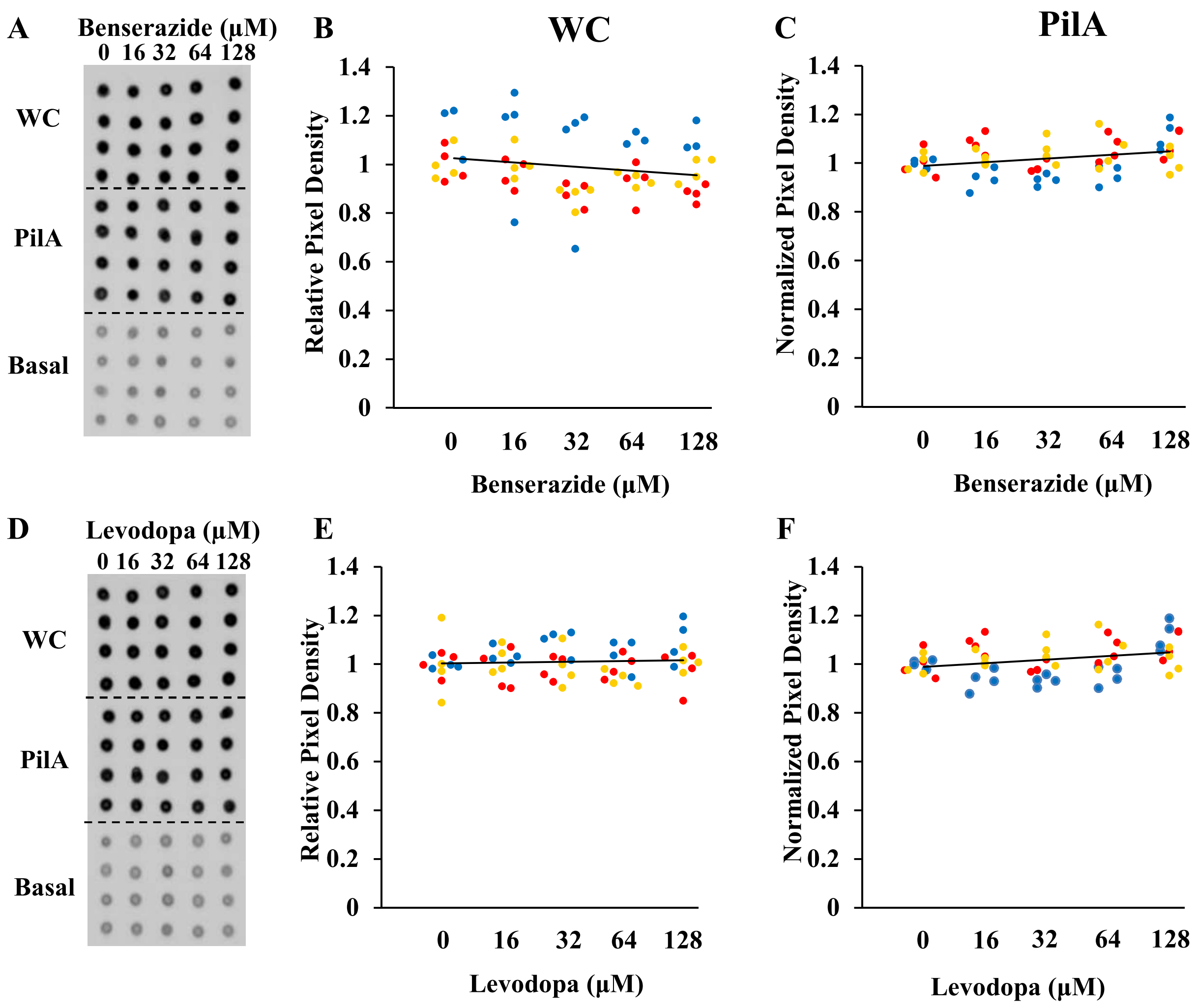

Supplemental Figure 6. Benserazide and levodopa do not alter the production of PilA in *M. xanthus*. Dot blot analysis of *M. xanthus* fractions treated with (**A**) benserazide or (**D**) levodopa using anti-pilin antibodies. The WC (whole cell) fraction is the lysate of cells with their T4P intact, the PilA fraction represents the internal PilA from cells with their T4P removed, while the "Basal" fraction represents the T4P present after the initial shearing prior to incubation with the compounds. Shown are single representatives from multiple experiments with similar results. Quantification of signals from the (**B** & **E**) WC (whole cell) and (**C** & **F**) PilA fractions of the dot blots with benserazide and levodopa. The pixel densities of the WC fractions were determined relative to that of the untreated samples. The pixel densities of the PilA fractions were normalized to the WC fraction. Shown are the results from three independent experiments, conducted in quadruplicate, each represented by circles of the same color. The linear trendline based on the averages is drawn for visualization purposes.

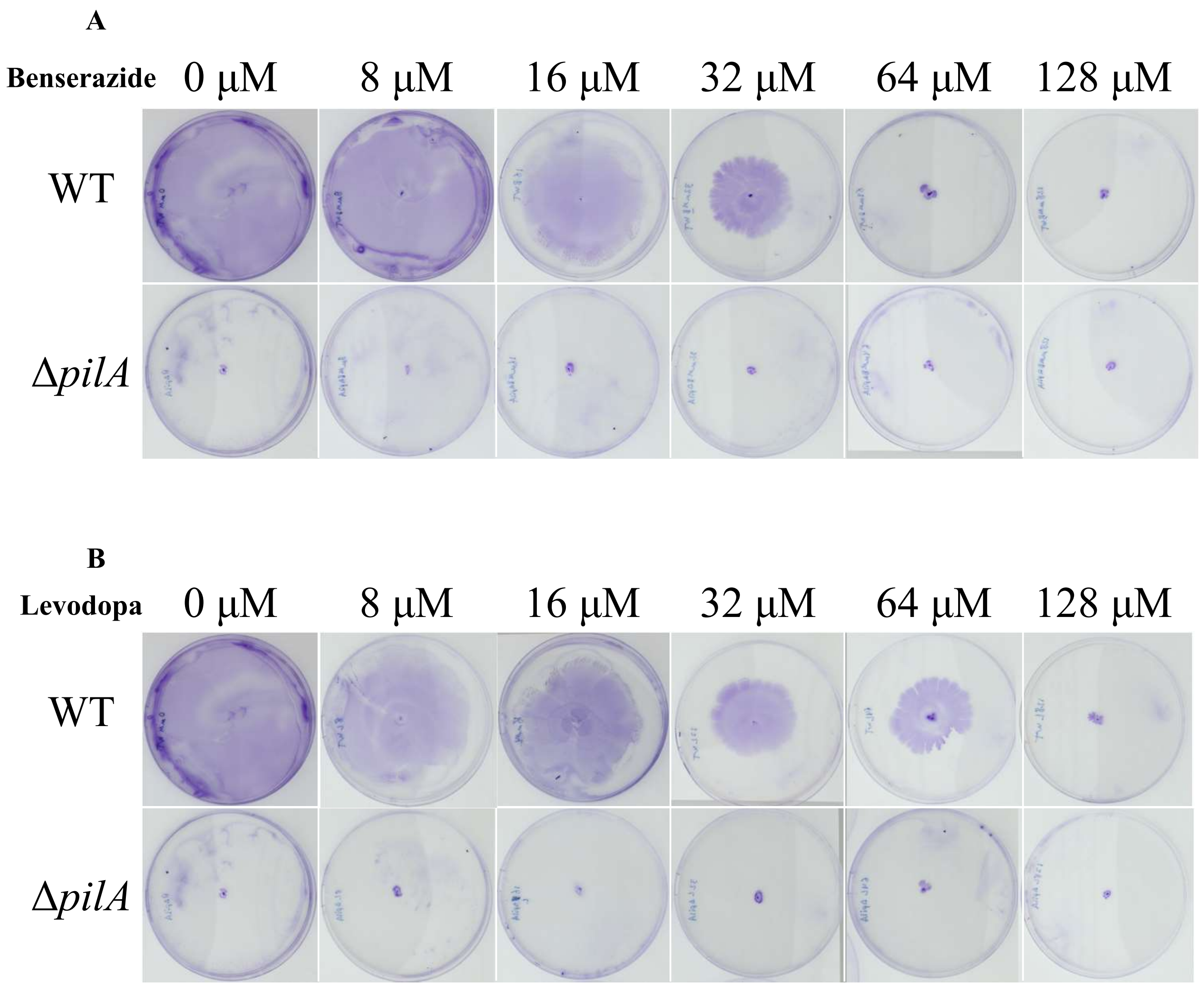

Supplemental Figure 7. Benserazide and levodopa inhibit the twitching motility of *A. nosocomialis*. Representative images of the twitching results treated with (**A**) benserazide or (**B**) levodopa. Single colonies of the WT and Δ*pilA A. nosocomialis* strains were stabbed through agar to inoculate the plates. Plates were incubated for three days at 37 °C. After incubation the agar was removed and adherent cells were stained with 1% crystal violet.
